# Methaemoglobinemia Induced by MDMA?

**DOI:** 10.1155/2011/494328

**Published:** 2011-10-19

**Authors:** L. L. W. Verhaert

**Affiliations:** Catharina Hospital Eindhoven, The Netherlands

## Abstract

*Case*. A 45-year-old man with a blank medical history presented at the emergency room with dizziness and cyanosis. Physical examination showed cyanosis with a peripheral saturation (SpO_2_) of 85%, he did not respond to supplemental oxygen. Arterial blood gas analysis showed a striking chocolate brown colour. Based on these data, we determined the arterial methaemoglobin concentration. This was 32%. We gave 100% oxygen and observed the patient in a medium care unit. The next day, patient could be discharged in good condition. Further inquiry about exhibitions and extensive history revealed that the patient used MDMA (3,4- methylenedioxymethamphetamine, the active ingredient of ecstasy). *Conclusion*. Acquired methaemoglobinemia is a condition that occurs infrequently, but is potentially life threatening. Different nutrients, medications, and chemicals can induce methaemoglobinemia by oxidation of haemoglobin. The clinical presentation of a patient with methaemoglobinemia is due to the impossibility of O_2_ binding and transport, resulting in tissue hypoxia. Important is to think about methaemoglobin in a patient who presents with cyanosis, a peripheral saturation of 85% that fails to respond properly to the administration of O_2_. Because methaemoglobin can be reduced physiologically, it is usually sufficient to remove the causative agent, to give O_2_, and to observe the patient.

## 1. Introduction

Methaemoglobinemia is a condition that occurs infrequently but is potentially life threatening. The aetiology may be congenital or acquired. Methaemoglobin is haemoglobin in which the iron molecule is oxidized. It loses the ability to bind O_2_, and the O_2_ affinity for each other haeme is raised, making the O_2_ dissociation curve shifts to the left which results in tissue hypoxia [1, 2].

Small amounts of methaemoglobin are produced physiologically, but the relative proportion of the total haemoglobin is kept constant to 1% of the total haemoglobin by physiological reduction (NADH-dependent methemoglobin reductase) [3].

The most frequent case reports of acquired methaemoglobinemia are caused by dapsone and local anaesthetics (lidocaine). We present a case of acquired methaemoglobinemia possibly induced by MDMA (3,4-methylenedioxymethamphetamine, the active ingredient of ecstasy).

## 2. Case

**Figure 1:**
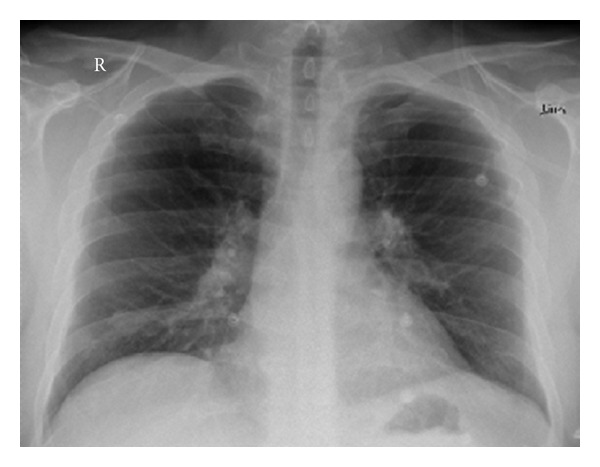
X-ray at admission.

A 45-year-old man with a blank medical history presented at the emergency room with dizziness and cyanosis. The discoloration was noticed by a colleague of him. On admission, he was well oriented in time and place. Physical examination showed cyanosis with a peripheral saturation (SpO_2_) of 85%, he did not respond to supplemental oxygen. He had a blood pressure of 140/70 mmHg, a pulse rate of 120/min, a SpO_2_ 87% with 15 litres of oxygen, and a temperature of 37.3°C. Arterial blood gas analysis showed a striking chocolate brown colour and a pH of 7.38, PO_2_ of 200 mmHg, PCO_2_ of 44 mmHg, and a HCO_3_ of 25.1 mmol/L. Chest X-Ray showed as a chance finding a mass on the right lung basis (this appeared to be a carcinoid after lobectomy later) (Figure 1). 

Based on these data, we determined the arterial methaemoglobin concentration. This was 32%. We gave 100% oxygen and observed the patient in a medium care unit. After one hour, the methaemoglobin concentration dropped to 23%. Another half hour later, the concentration was 16%. The next day, patient could be discharged in good condition.

Further inquiry about exhibitions and extensive history revealed that the patient used MDMA (3,4-methylenedioxymethamphetamine, the active ingredient of ecstasy). He used this more often, and after taking it, a blue discoloration of the hands and lips occurred but without vertigo and shortness of breath. At that night, he had used a large amount. Toxicological screening in urine showed >2000 micrograms per litre of amphetamine.

He did not use any other drug. He did use paracetamol for headache in big amounts. He did not eat or use products that can cause methaemoglobin (Table 3). Further investigations to congenital methaemoglobinemia were negative.

## 3. Conclusion

Acquired methaemoglobinemia is a condition that occurs infrequently, but is potentially life threatening. Different nutrients, medications, and chemicals can induce methaemoglobinemia by oxidation of haemoglobin. Most common causative agents are nitrates and nitrites, local anaesthetics, aniline and dapsone [1, 4, 5], Table 3.

The clinical, presentation of a patient with methaemoglobinemia is due to the impossibility of O_2_ binding and transport, resulting in tissue hypoxia [6].

Concentrations between 10% and 20% usually indicate cyanosis. This is mainly due to the methaemoglobin pigment and less because of deoxygenated haemoglobin. From a concentration of more than 20%, symptoms of respiratory distress, dizziness, headache, and fatigue develop. Lethargy and stupor origin from a concentration of 50% and death may occur from 70% of methaemoglobin concentration [1, 7], Table 1. 

The diagnosis is made difficult by the conventional pulse oximeter because it is unreliable in the presence of methaemoglobin. The saturation is calculated based on the ratio of light absorption at two wavelengths (660 and 940 nm). In contrast to oxy- and deoxyhaemoglobin, methaemoglobin absorbs both wavelengths similar. Because of this, there is always a saturation of about 85% measured, independent of O_2_ delivery. The arterial blood gas analysis suggests that the patient is well oxygenated because the PaO_2_ is based on a standard O_2_ dissociation curve. Often is given 100% oxygen with even high PaO_2_ values measured. The PO_2_ tells something about the delivery of oxygen from the lungs to blood, but in the case of methaemoglobin, this oxygen is not useful for tissue [2, 7]. Striking is the chocolate brown colour of the blood gas. The colour remains brown despite exposure to oxygen because of the inability to bind O_2_.

Important is to think about methaemoglobin in a patient who presents with cyanosis, a peripheral saturation of 85% that fails to respond properly to the administration of O_2_. 

For the treatment of acquired methaemoglobinemia, it is important to do a detailed history for medication use, diet, drinking water, and hobbies to discover the causative agent [2, 8]. Because methaemoglobin can be reduced physiologically, it is usually sufficient to remove the causative agent, to give O_2_, and to observe the patient.

At concentrations above 30%, it is generally recommended to administer methylene blue (1 to 2 mg/kg about 5 minutes, if necessary, repeat after 1 hour). Methylene blue acts as an exogenous electron acceptor for the NADPH methemoglobin reductase. Note that high doses of methylene blue may paradoxically induce methaemoglobinemia [4, 8]. It is contraindicated in glucose-6-phosphate dehydrogenase deficiency as methylene blue induces acute haemolysis. An alternative in this case is vitamin C 300 to 1000 mg per day orally.

At concentrations above 50%, it may be necessary to include patients in an intensive care for haemodialysis or plasmapheresis.

We believe that in this case report methaemoglobinemia was caused by MDMA (3,4-methylenedioxymethamphetamine, the active ingredient of ecstasy), given the high concentration of amphetamines in urine (>2000 micrograms per liter) at admission and the recurrent appearance of cyanosis after taking MDMA. In the literature, there is not any case report described so far. Biochemical acetaminophen may also cause methaemoglobinemia, but this is only in very large amounts described in animals [9].

**Figure 2:**
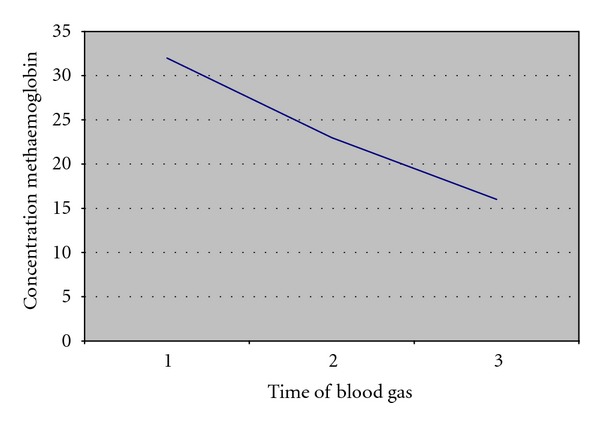
Concentration methaemoglobin over the time.

Methaemoglobin concentration at baseline was 32%. Since the patient had minimal complaints, we have given extra O_2_ and observed the patient in a medium care unit. We checked frequent arterial blood gases, Table 2. The methaemoglobin concentration after one hour had dropped to 23% (Figure 2). For this reason, we have not given IV methylene blue. The Patient could be discharged the next day.

This case illustrates the importance of thinking on the possibility of methaemoglobin. Since methaemoglobinemia is potentially life threatening, it is very important to discover the causative agents to avoid future problems.

## Figures and Tables

**Table 1:** Symptoms of methaemoglobinemia.

Methaemoglobin concentration	% Total haemoglobin	Symptoms
<1.5 g/dl	<10	None
1.5–3.0 g/dl	10–20	Cyanotic skin discoloration
3.0–4.5 g/dl	20–30	Anxiety, ligh headedness, headache, and tachycardia
4.5–7.5 g-dl	30–50	Fatigue, confusion, dizziness, tachypnea, and tachycardia
7.5–10.5 g/dl	50–70	Coma, seizures, arrythmias, and acidosis
>10.5 g/dl	>70	Death

**Table 2:** Blood gas analysis over the time.

Time	0	10 min later	1 hour later	1.5 hour later
O_2_ delivery	12 L O_2_	15L O_2_	15L O_2_	10L O_2_
Ph	7.38	7.39	7.39	7.41
PCO_2_ (mmHg)	44	44	43	43
HCO_3_ (mmol/L)	25.1	25.9	25.7	26.4
PO_2_ (mmHg)	200	240	250	300
Saturation (%)	ntb	97	97	98
Carboxyhaemoglobin (%)		0.20	−0.10	−0.10
Methaemoglobin (%)		32	23	16

**Table 3:** Causative agents of methaemoglobinemia.

Acetanilide	Hydroxylamine	Nitroprusside
Alloxan	Lidocaine	Paraquat/diquat
Aniline	Menadione	Phenacetin
Antipyrine	Metoclopramide	Phenazopyridine
Arsine	Methylene blue	Phenol
Benzene derivatives	Naphthalene	Phenylhydrazine
Benzocaine	Nitrates	Phenytoin
Chlorates	Nitroalkanes	Prilocaine
Chlorobenzene	Nitrochlorobenzene	Primaquine
Chloroquine	Nitrofuran	Smoke inhalation
Dapsone	Nitroglycerin	Sulphonamide antibiotics
Dinitrophenol		Trinitrotoluene
Dinitrotoluene		
